# Personality Traits and Higher-level Functional Capacity in Aging: Insights from a Japanese Older Population Study

**DOI:** 10.31662/jmaj.2024-0046

**Published:** 2024-08-23

**Authors:** Haruka Ishii, Yoko Ishii

**Affiliations:** 1 Academic Assembly Faculty of Medicine, University of Toyama, Toyama, Japan; 2 Graduate School of Health and Nutrition Sciences, The University of Nagano, Nagano, Japan

**Keywords:** Personality Traits, Higher-level Functional Capacity, Self-rated health, Japanese Elderly

## Introduction

In today’s aging population, we have a significant challenge in prolonging the older adult’s healthy lifespan. It is imperative to preserve physical independence and sustain functions equivalent to “instrumental independence” to ensure that older adults can maintain independence and lead active lives within their communities.

Higher-level functional capacity (HLFC) encompasses the intricate abilities outlined in Lawton’s final three stages (instrumental self-maintenance, intellectual activity, and social role performance) ^(1)^, with a scale developed in Japan to assess these stages ^(2)^. Typically, higher-level competencies decline before basic activities of daily living ^(2)^, making HLFC a valuable predictor of nursing care requirements and life expectancy. Similarly, Self-Rated Health (SRH) status demonstrates correlations with objective health indicators ^(3)^, serving as a reliable predictor of morbidity, mortality, and other health outcomes ^(4)^.

Personality traits reflect relatively stable tendencies to think, behave, and react in particular ways ^(5)^. Personality traits influence health behaviors throughout the lifespan, affecting mortality risk ^(6), (7)^. In a study exploring the correlation between personality traits and basic daily functioning among the elderly, high Neuroticism, low Extraversion, and low Conscientiousness were associated with a decline in physical independence ^(8)^. The relationship between HLFC and personality traits has been explored in Japan. Higher psychoticism and lower Extraversion are significantly associated with a risk of future functional decline among older adults in Iwate prefecture ^(9)^. However, nationwide surveys and gender differences in this context require further investigation.

Examining the relationship between personality traits, HLFC, and SRH in older adults could facilitate early detection and interventions targeting modifiable risk factors influencing individual aging differences. Such insights would prove valuable for healthcare professionals and families of older adults living together. Therefore, this study aimed to elucidate the impact of personality traits on HLFC and SRH among older adults in Japan, with a particular focus on gender differences.

## Materials and Methods

We utilized data from the “Survey of elderly people’s awareness regarding life management,” conducted by the Life Insurance Culture Center in October-November 2020. This survey used a sample selected through stratified, random, two-stage sampling to represent the Japanese population, selecting 3,000 individuals aged 60 or older (the first sampling unit: basic unit district set at the time of the 2015 census; the target person extraction: Basic Resident Register). The data were collected using the placement method, with professional staff conducting face-to-face interviews with the respondents. A total of 2083 participants were included; after excluding missing data, 1859 respondents provided sufficient data for further analysis. We employed questions on personality traits, HLFC, and SRH from the survey instrument.

Personality traits were assessed using the TIPI-J ^(10)^, the Japanese adaptation of the Ten Item Personality Inventory (TIPI) ^(11)^. TIPI is a concise version of the Five-Factor Model, which has amassed extensive empirical support for a global understanding of personality traits ^(5)^. The Five-Factor Model categorizes personality into five overarching dimensions: Extraversion, Agreeableness, Conscientiousness, Neuroticism, and Openness (openness to experience). The TIPI is applied across various domains, such as social psychology, political psychology, and behavioral economics.

HLFC was evaluated using the Tokyo Metropolitan Institute of Gerontology Index of Competence (TMIG-IC) ^(2)^, a multidimensional assessment tool comprising 13 items categorized into three subscales: instrumental self-maintenance (5 items), intellectual activity (4 items), and social role (4 items).

SRH was assessed based on the question: “How would you rate your general health status?”

Univariate analysis was conducted using the chi-square test. For multivariate analysis, binomial logistic regression analyses using a forced input method were performed to analyze factors influencing changes in HLFC and SRH and to calculate the odds ratio and 95% confidence interval (CI) after adjusting for age, gender, living alone, household financial assets, city size, and residential areas. In the binomial logistic regression model, we dichotomized the variables into two categories: less than the median and equal to or greater than the median.

All data were analyzed using SPSS version 28.0 (IBM Japan, Ltd., Tokyo, Japan), with a significance level set at 0.05.

Ethics approval was deemed unnecessary since this study was a secondary analysis of publicly available data from the University of Tokyo’s Center for Social Research and Data Archives, Institute of Social Science.

## Results

Table 1 presents the participants’ demographic characteristics and the distributions of general characteristics, each personality trait, HLFC, and SRH.

**Table 1.** Demographic Characteristics.

		Number	%			
The study participants	Total	1859				
	Men	857	46.1
	Women	1002	53.9
						
		Mean	S.D.	Median	Minimum	Maximum
Age	Men	72.41	7.71	71	60	94
	Women	72.62	7.74	72	60	98
						
		Mean	S.D.	Median	Minimum	Maximum
Five-factor model of personality	Extraversion	8.60	2.27	8	2	14
	Agreeableness	10.68	1.85	11	3	14
	Conscientiousness	9.18	2.11	9	2	14
	Neuroticism	7.31	2.13	8	2	14
	Openness	7.82	2.15	8	2	14
Higher-level functional capacity	Instrumental self-maintenance	4.78	0.80	5	0	5
	Intellectual activity	3.54	0.82	4	0	4
	Social role	3.23	1.08	4	0	4
	Total score of higher-level functional capacity	11.57	2.09	12	0	13
Self-rated health		3.59	1.04	3	1	5
						
		**Number**	**%**			
Household financial assets	Less than 1 million yen	267	14.4			
	1 million−10 million yen	482	25.9			
	10 million−20 million yen	255	13.7			
	20 million−50 million yen	288	15.5			
	50 million−100 million yen	111	6.0			
	100 million yen or more	23	1.2			
	No answer	433	23.3			
Living alone	No	1578	84.9
	Yes	281	15.1
City size	Big City	637	34.3
	Cities with a population of 100,000 or more	756	40.7
	Cities with a population of less than 100,000	320	17.2
	Rural districts	146	7.9
Residential area	Tokyo	405	21.8
	Aichi	255	13.7
	Osaka	289	15.5
	Hokkaido	56	3.0
	Tohoku	85	4.6
	Kanto (Excluding Tokyo)	266	14.3
	Hokuriku	56	3.0
	Chubu (Excluding Aichi)	92	4.9
	Kinki (Excluding Osaka)	101	5.4
	Chugoku	84	4.5
	Shikoku	39	2.1
	North Kyushu	79	4.2
	South Kyushu	52	2.8

Table 2 displays the associations between each personality trait and the total scores of HLFC and between each personality trait and SRH.

**Table 2.** The Associations between Personality Traits and Higher-Level Functional Capacity.

			**Higher-level functional capacity**		**Univariate analysis**			**Multivariate analysis**		
			**Low (0-11)**	**Median/High (12-13)**	**OR**	**95% CI**	**p-value**	**AOR ǂ**	**95% CI**	**p-value**
Extraversion	Men	Low (2-7)	149	130	2.09	1.56-2.79	< 0.001***	2.08	1.56-2.78	< 0.001***
		Median/High (8-14)	205	373						
	Women	Low (2-7)	107	149	2.00	1.49-2.69	< 0.001***	2.02	1.48-2.73	< 0.001***
		Median/High (8-14)	197	549						
	All	Low (2-7)	256	279	2.10	1.71-2.59	< 0.001***	2.06	1.67-2.54	< 0.001***
		Median/High (8-14)	402	922						
Agreeableness	Men	Low (3-10)	183	246	1.12	0.85-1.47	0.421	1.13	0.86-1.49	0.382
		Median/High (11-14)	171	257						
	Women	Low (3-10)	134	265	1.29	0.98-1.69	0.069	1.37	1.03-1.81	0.029*
		Median/High (11-14)	170	433						
	All	Low (3-10)	317	511	1.26	1.05-1.52	0.019*	1.24	1.02-1.51	0.029*
		Median/High (11-14)	341	690						
Conscientiousness	Men	Low (2-8)	71	72	1.50	1.05-2.15	0.026*	1.51	1.14-2.00	0.004*
		Median/High (9-14)	283	431						
	Women	Low (2-8)	79	115	1.78	1.29-2.04	< 0.001***	1.61	1.22-2.14	< 0.001***
		Median/High (9-14)	225	583						
	All	Low (2-8)	150	187	1.60	1.26-2.04	< 0.001***	1.54	1.66-1.88	< 0.001***
		Median/High (9-14)	508	1014						
Neuroticism	Men	Low (2-7)	172	275	0.78	0.60-1.03	0.079	0.78	0.60-1.03	0.081
		Median/High (8-14)	182	228						
	Women	Low (2-7)	113	341	0.62	0.47-0.82	< 0.001***	0.59	0.45-0.78	< 0.001***
		Median/High (8-14)	191	357						
	All	Low (2-7)	285	616	0.73	0.60-0.88	< 0.001***	0.70	0.57-0.84	< 0.001***
		Median/High (8-14)	373	585						
Openness	Men	Low (1-2)	146	140	1.82	1.36-2.42	< 0.001***	1.81	1.36-2.41	< 0.001***
		Median/High (3-5)	208	363						
	Women	Low (1-2)	157	314	1.31	0.99-1.71	0.052	1.23	0.93-1.62	0.150
		Median/High (3-5)	147	384						
	All	Low (1-2)	303	454	1.40	1.16-1.70	0.001**	1.5	1.23-1.83	< 0.001***
		Median/High (3-5)	355	747						
										
			**Higher-level functional capacity**		**Univariate analysis**			**Multivariate analysis**		
			**Low (0-11)**	**Median/High (12-13)**	**OR**	**95% CI**	**p-value**	**AOR ǂ**	**95% CI**	**p-value**
Self-rated health	Men	Low (1-2)	73	45	2.64	1.77-3.95	< 0.001***	2.69	1.79-4.04	< 0.001***
		Median/High (3-5)	281	458						
	Women	Low (1-2)	63	61	2.73	1.86-4.00	< 0.001***	2.14	1.44-3.19	< 0.001***
		Median/High (3-5)	241	637						
	All	Low (1-2)	136	106	2.69	2.05-3.54	< 0.001***	2.45	1.85-3.25	< 0.001***
		Median/High (3-5)	522	1095						
										
			**Self-rated health**		**Univariate analysis**			**Multivariate analysis**		
			**Low (1-2)**	**Median/High (3-5)**	**OR**	**95% CI**	**p-value**	**AOR ǂ**	**95% CI**	**p-value**
Extraversion	Men	Low (2-7)	48	231	1.51	1.01-2.25	0.043*	1.55	1.03-2.33	0.035*
		Median/High (8-14)	70	508						
	Women	Low (2-7)	37	219	1.28	0.85-1.94	0.241	1.24	0.80-1.91	0.339
		Median/High (8-14)	87	659						
	All	Low (2-7)	85	450	1.40	1.06-1.87	0.019*	1.40	1.05-1.89	0.024*
		Median/High (8-14)	157	1167						
Agreeableness	Men	Low (3-10)	72	357	1.67	1.13-2.49	0.104	1.87	1.24-2.81	0.003**
		Median/High (11-14)	46	382						
	Women	Low (3-10)	51	348	1.06	0.73-1.56	0.750	1.16	0.78-1.72	0.468
		Median/High (11-14)	73	530						
	All	Low (3-10)	123	705	1.34	1.02-1.75	0.035*	1.46	1.11-1.94	0.008**
		Median/High (11-14)	119	912						
Conscientiousness	Men	Low (2-8)	33	110	2.22	1.42-3.48	< 0.001***	1.76	1.18-2.64	0.006**
		Median/High (9-14)	85	629						
	Women	Low (2-8)	23	171	0.94	0.58-1.53	0.807	1.50	1.01-2.24	0.044*
		Median/High (9-14)	101	707						
	All	Low (2-8)	56	281	1.43	1.03-1.98	0.030*	1.62	1.22-2.15	< 0.001***
		Median/High (9-14)	186	1336						
Neuroticism	Men	Low (2-7)	50	397	0.63	0.43-0.94	0.021*	0.64	0.43-0.95	0.027*
		Median/High (8-14)	68	342						
	Women	Low (2-7)	43	411	0.60	0.41-0.89	0.011*	0.54	0.36-0.82	0.003**
		Median/High (8-14)	81	467						
	All	Low (2-7)	93	808	0.62	0.47-0.82	0.001**	0.59	0.45-0.79	< 0.001***
		Median/High (8-14)	149	809						
Openness	Men	Low (1-2)	54	232	1.84	1.24-2.73	0.002**	1.94	1.30-2.91	0.001**
		Median/High (3-5)	64	507						
	Women	Low (1-2)	65	406	1.28	0.88-1.87	0.197	1.14	0.77-1.68	0.529
		Median/High (3-5)	59	472						
	All	Low (1-2)	119	638	1.58	1.13-1.95	0.004**	1.45	1.12-1.98	0.006**
		Median/High (3-5)	123	979						

*P < 0.05, ** P < 0.01, *** P < 0.0001, AOR: adjusted odds ratio, OR: odds ratio, CI: confidence interval ǂ: All: Adjusted for age, gender, living alone, household financial assets, city size, and residential area. ǂ: Men and Women: Adjusted for age, living alone, household financial assets, city size, and residential area.

In the multivariate analyses by gender, Extraversion and Conscientiousness exhibited positive associations with HLFC in men (OR = 2.08; 95% CI: 1.56-2.78; p < 0.001 and OR = 1.51; 95% CI: 1.14-2.00; p = 0.004, respectively) and women (OR = 2.02; 95% CI: 1.48-2.73; p < 0.001 and OR = 1.61; 95% CI: 1.22-2.14; p < 0.001, respectively). Notably, Openness was positively associated with HLFC in men (OR = 1.81; 95% CI: 1.36-2.41; p < 0.001) but not in women (OR = 1.23; 95% CI: 0.93-1.62; p = 0.150). Conversely, Neuroticism was negatively associated with HLFC in women (OR = 0.59; 95% CI: 0.45-0.78; p < 0.001) but not in men (OR = 0.78; 95% CI: 0.60-1.03; p = 0.081). In the multivariate analysis overall, Extraversion, Conscientiousness, Openness, and Agreeableness were positively associated with HLFC (OR = 2.06; 95% CI: 1.67-2.54; p < 0.001, OR = 1.54; 95% CI: 1.66-1.88; p < 0.001, OR = 1.50; 95% CI: 1.23-1.83; p < 0.001, and OR = 1.24; 95% CI: 1.02-1.51; p = 0.029, respectively), while Neuroticism was negatively associated with HLFC (OR = 0.70; 95% CI: 0.57-0.84; p < 0.001).

In the multivariate analysis by gender, SRH demonstrated a positive association with HLFC in both men (OR = 2.69; 95% CI: 1.79-4.04; p < 0.001) and women (OR = 2.14; 95% CI: 1.44-3.19; p < 0.001), as observed in Table 2. The multivariate analysis also confirmed this association overall (OR = 2.45; 95% CI: 1.85-3.25; p < 0.001).

In the multivariate analysis by gender, Conscientiousness demonstrated a positive association with SRH in men (OR = 1.76; 95% CI: 1.18-2.64; p < 0.001), women (OR = 1.50; 95% CI: 1.01-2.24, p = 0.044), and overall (OR = 1.62; 95% CI: 1.22-2.15; p < 0.001). Openness, Agreeableness, and Extraversion exhibited positive associations with SRH in men (OR = 1.94; 95% CI: 1.30-2.91; p = 0.001, OR = 1.87; 95% CI: 1.24-2.81; p = 0.003, and OR = 1.55; 95% CI: 1.03-2.33; p = 0.035, respectively) but not in women (OR = 1.14; 95% CI: 0.77-1.68; p = 0.529, OR = 1.16, 95% CI: 0.78-1.72; p = 0.468, and OR = 1.24; 95% CI: 0.80-1.91; p = 0.339, respectively). Neuroticism was negatively associated with SRH in men (OR = 0.64; 95% CI: 0.43-0.95; p = 0.027) and women (OR = 0.54; 95% CI: 0.36-0.82; p = 0.003). In multivariate analysis overall, Conscientiousness, Openness, Agreeableness, and Extraversion were positively associated with SRH (OR = 1.62; 95% CI: 1.22-2.15; p < 0.001, OR = 1.45; 95% CI: 1.12-1.98; p = 0.006, OR = 1.46; 95% CI: 1.11-1.94; p = 0.008, and OR = 1.40; 95% CI: 1.05-1.89; p = 0.024, respectively), whereas Neuroticism was negatively associated with SRH (OR = 0.59; 95% CI: 0.45-0.79; p < 0.001).

## Discussion

The present study revealed that higher levels of Extraversion, Conscientiousness, Openness, and Agreeableness were associated with higher scores of HLFC in the older adults, while Neuroticism was linked to poorer HLFC. Noteworthy was the significantly positive association of Openness in men, contrasted with the significantly negative association of Neuroticism in women.

In the present study, Extraversion emerged as the most significantly associated personality trait with HLFC in men and women among the five factors. Extraversion is characterized by social activity, assertiveness, positive emotionality, high activity levels, and sensitivity to reward ^(12)^. Individuals who score high in Extraversion are more inclined to engage in social activities, which may indirectly contribute to their higher functional capacity.

In this study, the positive influence of Conscientiousness on HLFC was more pronounced in women than in men, whereas its effect on SRH was more prominent in men than in women. A meta-analysis comprising 194 reports examining Conscientiousness and prominent behavioral contributors to mortality found that Conscientiousness was negatively associated with all risky behaviors and positively associated with all health-promoting behaviors ^(13)^.

Furthermore, the positive influence of Openness on both HLFC and SRH was significant in men but not in women. Openness to experience, which is highly correlated with education, may be linked to HLFC through intellectual activity and social roles ^(7)^.

Additionally, the negative influence of Neuroticism on HLFC was more pronounced in women than in men, although its effect on SRH was almost similar between men and women. Individuals high in Neuroticism are more likely to experience greater negative effects and daily stress ^(14)^. Since stress is associated with unhealthy behaviors, such as unhealthy dietary patterns and difficulty sleeping, individuals experiencing stress might be at an increased risk of morbidity.

This study has several limitations. First, it may not be representative of the entire Japanese older population. However, the distribution of participants by prefecture in this study closely matches the population distribution in Japan. Second, due to data limitations, the multivariate analysis did not include some potentially influential factors, such as educational level and comorbid health conditions.

In addition to maintaining HLFC, factors contributing to an extended cognitive health span are crucial for an aging population. In a secondary data analysis of the Rush Memory and Aging Project spanning up to 23 annual assessments, personality traits were particularly significant in the transition from no cognitive impairment (NCI) to mild cognitive impairment (MCI) ^(7)^. Notably, higher Conscientiousness and lower Neuroticism were significantly associated with a decreased risk of transitioning from NCI to MCI. In contrast, higher Extraversion was associated with the transition from MCI to NCI. This association may reflect the increased social engagement and support characteristics of individuals with high Extraversion ^(7)^.

Although further examination is necessary to elucidate the associations between personality traits and health outcomes, a better understanding of personality traits could be useful for the early detection and intervention of older individuals at high risk. Knowledge of personality-driven health behaviors may provide useful insights for developing more effective health promotion and disease prevention interventions. We should tailor intervention programs to individual differences, considering the ethical implications of personality assessments.

## Article Information

### Conflicts of Interest

None

### Sources of Funding

This work was supported by JSPS KAKENHI grant number JP22K02139.

### Acknowledgement

The data for this secondary analysis, “Survey of elderly people’s awareness regarding life management,” conducted by the Life Insurance Culture Center, was provided by the Social Science Japan Data Archive, Center for Social Research and Data Archives, Institute of Social Science, The University of Tokyo.

### Author Contributions

HI: data analysis and manuscript writing; YI: project development, data management, data analysis, and manuscript writing/editing.
